# Efficacy and safety of interim oncology treatments introduced for solid cancers during the COVID-19 pandemic in England: a retrospective evidence-based analysis

**DOI:** 10.1016/j.lanepe.2024.101062

**Published:** 2024-09-10

**Authors:** Mark P. Lythgoe, Alica-Joana Emhardt, Huseyin Naci, Jonathan Krell, Richard Sullivan, Ajay Aggarwal

**Affiliations:** a Department of Surgery & Cancer, Imperial College London, London, UK; b Department of Health Services Research & Policy, London School of Hygiene & Tropical Medicine, London; Department of Medicine III, LMU University Hospital, LMU Munich, Munich, Germany; c Department of Health Policy, London School of Economics and Political Science, London, UK; d King's College London, Institute of Cancer Policy, London, UK

**Keywords:** Cancer, COVID-19, NICE, Immunosuppression, Immunotherapy, Immune checkpoint inhibitors

## Abstract

**Background:**

The COVID-19 global pandemic placed unprecedented pressure on cancer services, requiring new interim Systemic Anti-Cancer Treatments (SACT) options to mitigate risks to patients and maintain cancer services. In this study we analyse interim COVID-19 SACT therapy options recommended in England, evaluating the evidence supporting inclusion and delineating how these have been integrated into routine cancer care.

**Methods:**

We performed a retrospective analysis of interim Systemic Anti-Cancer Treatments endorsed by NHS England during the COVID-19 pandemic. Interim therapy options were compared to baseline (replacement) therapies by comparing data from the key pivotal trial(s) in terms of clinical efficacy and potential benefits (e.g., reduced immunosuppression or improved adverse effect profile) within the context of the pandemic. Furthermore, we evaluated the evolution of these interim SACT options, exploring if these have been integrated into current treatment pathways or are no longer accessible at the pandemic end.

**Findings:**

31 interim oncology treatment options, across 36 indications, for solid cancers were endorsed by NHS England between March 2020 and August 2021. Interim therapies focused on the metastatic setting (83%; 30/36), allowing greater utilisation of immune checkpoint inhibitors (45%; 14/31) and targeted therapies (26%; 8/31), in place of cytotoxic chemotherapy. Overall, 36% (13/36) of therapies could not have efficacy compared with baseline treatments due to a paucity of evidence. For those which could, 39% (9/23) had superior efficacy (e.g., overall survival), 26% (6/23) had equivocal efficacy and 35% (8/23) lower efficacy. 53% (19/36) of interim therapies had better or equivocal toxicity profiles (when assessable), and/or were associated with reduced immunosuppression. Almost half (47%; 17/36) of interim therapies did not have UK market authorisation, being classified as ‘off label’ use. Analysing access to interim options at the end of the pandemic (May 2023) identified 19 (53% 19/36) interim options were fully available, and a further four (11% 4/36) therapies were partially available.

**Interpretation:**

Interim SACT options, introduced in England, across a range of solid cancers supported delivery of cancer services during the pandemic. Most interim therapies did not demonstrate superior efficacy, but provided other important benefits (e.g., reduced immunosuppression) in the context of the pandemic.

**Funding:**

None.

**Research in context**

**Evidence before this study**

Due to the rapid emergence of SARS-CoV-2 in 2020 there was a rapid decline in prescribing of Systemic Anti-Cancer Treatments in England, due primarily to concerns around immunosuppression. In England, new interim treatments were recommended by NHS England and NICE, in place of standard-of-care options, to facilitate cancer treatment during the pandemic based on recommendations of a Chemotherapy Clinical Reference Group. Previous evidence has demonstrated a high uptake of these interim therapy recommendations, reversing declines in anti-cancer prescribing at the start of the pandemic. However, the evidence supporting these recommendations (including many ‘off label’ uses) has not been published.

Therefore, we searched PubMed for pivotal studies (search term in Appendix) for each interim therapy recommendation (Table 3), and baseline (replacement) therapy to identify the evidence supporting these. We identified comparative efficacy for 23 (64%; 23/36) interim COVID-19 treatment options However, for 13 (36%) interim COVID-19 treatment options no comparative efficacy could be identified.

**Added value of this study**

To our knowledge, this is the first study to assess the evidence base for interim therapy recommendations made by NHS England and NICE during the COVID-19 pandemic, transforming the management of many cancers during this challenging period. We identified 31 new interim Systemic Anti-Cancer Treatments (across 36 indications) endorsed by NHS England for the treatment of solid cancer(s) during the COVID-19 pandemic. Most interim therapy options advocated switching to less toxic and immunosuppressive therapies (e.g., cytotoxic chemotherapy to immune checkpoint inhibitors). Only 64% of interim therapy options had direct evidence supporting their use, in place of baseline treatments. 35% of interim therapies involved switching to less efficacious options, and 47% were not licensed, requiring ‘off-label’ use, increasing prescriber responsibility. At the end of the pandemic only 53% of interim therapies remain fully available, with many being withdrawn from use.

**Implications of all the available evidence**

In England interim therapy options introduced by NHS England and NICE during the COVID-19 pandemic reversed declines in the prescribing of Systemic Anti-Cancer Treatments at the start of pandemic. However, only two-thirds of these interim options had published evidence supporting their use in place of standard of care treatments. Our work highlights the need to put in place robust advisory and legislative frameworks to ensure the continuance and viability of cancer treatment in England in the face of any future global healthcare crisis. Clinical outcomes of patients receiving interim SACT therapies needs to be collected to inform about the viability, and success, of this strategy. Furthermore, this work has implications beyond the national level, and can be used as evidence to guide international stakeholders to allow for the global harmonisation of cancer management when faced with healthcare emergencies.

## Introduction

The emergence of severe acute respiratory syndrome coronavirus 2 virus (SARS-CoV-2) in 2019, and the subsequent COVID-19 pandemic, placed immeasurable strain on global healthcare systems.^1^ It was recognised early in the pandemic, cancer patients exposed to SARS-CoV-2 virus had a high likelihood of serious clinical sequelae (e.g., respiratory failure), with estimates of 30-day all-cause mortality ranging from 13% to 57%, due to advanced age, likelihood of significant co-morbidities and the deleterious effects of concurrent malignancy, and the anti-cancer therapy, on overall immune functioning.^2^, ^3^, ^4^ This effect was most pronounced during the early phase of the pandemic, before the advent of effective vaccines and pharmacotherapy.^5^, ^6^, ^7^

The substantial reduction in UK oncology services during the COVID-19 pandemic, necessitated prioritisation of resources to areas deemed essential and/or offering highest patient benefit. Guidance (March 2020) from the National Health Service (NHS) England and the National Institute for Health and Care Excellence (NICE) aided clinical decisions by permitting prioritisation of Systemic Anti-Cancer Treatments (SACT) delivery based on evidence level (Table 1), from level 1 (high priority), for curative treatments (e.g., BEP chemotherapy in germ cell cancers), to level 6 (low priority) for treatments with a <50% chance of palliation and <1 years life extension (e.g., trifluridine/tipiracil in advanced colorectal cancer)).^8^, ^9^, ^10^ This facilitated cancer services prioritisation according to potential benefit, an approach replicated by many countries and international advisory bodies (e.g., European Society of Medical Oncology).^11^

**Table 1:** Categorisation of Systemic Anti-Cancer Therapies (SACT) based on treatment intent and risk-benefit ratio (adapted from NICE guideline [NG161]).

Priority	Prioritising patients for SACT
Level 1	• Curative therapy with a high (>50%) chance of success • Adjuvant or neoadjuvant treatment which adds at least 50% chance of cure to surgery or radiotherapy alone or treatment given at relapse
Level 2	• Curative therapy with an intermediate (15–50%) chance of success • Adjuvant or neoadjuvant treatment which adds 20%–50% chance of cure to surgery or radiotherapy alone or treatment given at relapse
Level 3	• Curative therapy with a low (10%–20%) chance of success • Adjuvant or neoadjuvant treatment which adds 10%–20% chance of cure to surgery or radiotherapy alone or treatment given at relapse • Non-curative treatment with a high (more than 50%) chance of more than 1 year extension to life
Level 4	• Curative therapy with a very low (0%–10%) chance of success • Adjuvant or neoadjuvant treatment which adds less than 10% chance of cure to surgery or radiotherapy alone or treatment given at relapse • Non-curative treatment with an intermediate (15%–50%) chance of more than 1 year extension to life
Level 5	• Non-curative therapy with a high (>50%) chance of palliation/temporary tumour control but <1 year expected life extension.
Level 6	• Non-curative therapy with an intermediate (15–50%) chance of palliation or temporary tumour control and <1 year life extension

Patients at highest risk of severe clinical sequelae, or death, linked to COVID-19 included those with incurable cancer (stage 4 disease), lung cancer (any stage), and was further compounded by significant comorbidities (e.g., cirrhosis) and older age (>75 years).^12^, ^13^, ^14^, ^15^ Early studies suggested a strong association between cytotoxic chemotherapy and severe COVID-19 sequelae, when compared to non-cytotoxic SACT (e.g., immune checkpoint inhibitors).^16^^,^^17^ However, it is now appreciated the urgency with which large retrospective cohort data was collected led to significant limitations (e.g., short follow-up, missing data), likely overestimated this risk significantly.^18^^,^^19^ Nevertheless, the oncology community needed to protect a population assumed highly vulnerable. Therefore, interim cancer treatment options were introduced by NHS England (Appendix), bypassing routine NHS and NICE processes (pre-pandemic) for new cancer drug approval, endorsing interim therapies, some of which were ‘off label’, to permit greater flexibility in cancer management during the pandemic, and ensuring clinicians had additional treatment options.^10^^,^^20^ Interim therapy options permitted alteration, or replacement, to the current SACT treatment recommendations, offering benefits in terms of resource delivery and/or COVID-19 related benefits (e.g., reduced immunosuppression). However, it remains unknown what evidence was used to support introduction of interim treatment options, and the value they added, especially in the context of the pandemic.

Interim treatment options were selected based on recommendations of the Chemotherapy Clinical Reference Group (CCRG), consisting of oncologists, specialist pharmacists, chemotherapy nurses and patient and public voice representatives, being endorsed by NHS England and NHS Improvement. Each interim treatment option was clinically assessed against five criteria (Table 2). For inclusion, a therapy must have met specification 4 (feasible to deliver) and 5 (adequate capacity to deliver), and at least one additional criterion from the following three specifications; reduced immunosuppressive (specification 1), administered at home or likely to result in less exposure to COVID-19 (specification 2) and/or be less resource intense (specification 3). It is unknown if interim therapies recommended, adhered to these, and/or focused on particular specifications.

**Table 2:** Selection criteria for interim treatment options during the COVID-19 pandemic (adapted from NICE guideline [NG161]).

	Specification
Criteria 1	Treatment is less immunosuppressive and thereby mitigates a patient's likelihood of contracting COVID-19 or becoming seriously ill from COVID-19
Criteria 2	Treatment can be administered at home or in a setting that reduces the patient's exposure to COVID-19
Criteria 3	Treatment is less resource intensive and makes better use of clinical capacity
Criteria 4	Treatment is feasible; that is, it is not likely to require significant service change or additional training
Criteria 5	Likely to be adequate capacity in the relevant sector (such as home care providers) to deliver the treatment.

Interim treatment options selected must meet criteria 1 or 2 or 3. In addition all treatments must meet **both** criteria 4 and 5 for selection.

**Table 3:** NHS England interim treatment options during the COVID-19 pandemic (adapted from NICE Guidelines [NG161] (October 2022)).

Drug (Cancer Type)	COVID interim indication	Selection criteria^p^	Comparator trial	Comparative efficacy	Safety profile		COVID priority	UK license	Relevant NICE recommendation(s) at date of censoring^m^ [Refers to specific assessment number]
					All grade 3 or 4 toxicity rate	Neutropenia grade 3 or 4 toxicity rate			
**Atezolizumab** (Urothelial Cancer)	First-line immunotherapy instead of chemotherapy	1 + 3	**IMvigor130** (Group B & Group C comparison): Atezolizumab with or without chemotherapy in metastatic urothelial cancer	2.6-month gain in OS	44% Reduction	30% Reduction	Level 3	Cisplatin ineligible, and whose tumours have a PD-L1 expression ≥5%	• [TA739] First line in cisplatin ineligible patients if PD-L1 expression ≥5% (partial approval)
**Bisphosphonates** (Breast Cancer)	Suspend treatment with adjuvant bisphosphonates (e.g., zoledronic acid or sodium clodronate)	2 + 3	Early Breast Cancer Trialists' Collaborative Group Meta-Analysis	No statistically significant DFS difference	Not assessed in meta-analysis		Level 4	Unlicensed	• [NG101] Adjuvant treatment for postmenopausal women with node-positive or node-negative with high risk of reoccurrence (full approval)
**Trastuzumab** (Breast Cancer)	Reduced course of adjuvant treatment from 12 months to 6 months	3	**PERSEPHONE:** 6 versus 12 months of adjuvant trastuzumab for HER2-positive early breast cancer	Non-inferiority demonstrated between 6- and 12-months treatment (at 5-years)	5% Reduction	NR	Level 3	Adjuvant treatment with HER2 positive early breast cancer	• [NG101] Adjuvant trastuzumab for HER2 positive breast cancer, for 1 year (partial approval)
**Pertuzumab + Trastuzumab** (Breast Cancer)	Neo-adjuvant therapy, adjuvant therapy, locally recurrent or metastatic disease without chemotherapy	1	**NeoSphere** (Neoadjuvant; Group B & Group C): Efficacy and safety of neoadjuvant pertuzumab and trastuzumab in women with locally advanced, inflammatory, or early HER2-positive breast cancer	29% reduction in pCR without chemotherapy	6% Reduction	44% Reduction	Level 2	In combination with chemotherapy in neoadjuvant treatment of HER2-positive breast cancer	• [TA424] Pertuzumab, in combination with trastuzumab and chemotherapy for neoadjuvant treatment of HER2 positive breast cancer (no approval)
		3	**PERNETTA** (Metastatic): A non-comparative randomized open label phase II trial of pertuzumab + trastuzumab with or without chemotherapy both followed by T-DM1 in case of progression	14.9-month reduction in PFS	Not Reported		Level 5	In combination with docetaxel in HER2-positive metastatic or breast cancer, who have not received previous anti-HER2 therapy or chemotherapy	• [TA509] Pertuzumab, with trastuzumab and docetaxel for treating HER2 positive breast cancer (no approval)
**Capecitabine** (Breast Cancer)	Switch to oral capecitabine from intravenous taxanes with anti-HER2 therapies for metastatic disease	1 + 2	Randomised, phase II trial comparing oral capecitabine with paclitaxel in patients with metastatic/advanced breast cancer^a^ pre-treated with anthracyclines	10% improvement in ORR	35% Reduction	44% Reduction	Level 6	**Two Relevant licensed indications:** • In combination with docetaxel for patients with metastatic breast cancer after failure of cytotoxic chemotherapy (anthracycline); • As monotherapy for the treatment of or metastatic breast cancer after failure of taxanes and an anthracycline-containing chemotherapy regimen	• [CG81] patients with breast cancer unsuitable for anthracyclines systemic chemotherapy should be offered 2nd line: single-agent vinorelbine or capecitabine (after docetaxel) (no approval)
**Abraxane** (Breast Cancer)	Substitute albumin-bound paclitaxel (Abraxane) for paclitaxel	1	CA012: Phase III Trial of Nanoparticle Albumin-Bound Paclitaxel Compared With Polyethylated Castor Oil–Based Paclitaxel in Women With Breast Cancer	14% Improvement in ORR	Reported as similar	12% Reduction	Level 4	Monotherapy treatment of metastatic breast cancer in adult patients who have failed first-line treatment for metastatic disease and for whom standard, anthracycline containing therapy is not indicated	• Available via CDF if being switched to nab-paclitaxel from either paclitaxel or docetaxel either following a severe hypersensitivity reaction which precludes further exposure (partial approval)
**Atezolizumab** (Breast Cancer)	Use in triple negative metastatic breast cancer instead of chemotherapy	1	Long-term Clinical Outcomes and Biomarker Analyses of Atezolizumab Therapy for Patients With Metastatic Triple-Negative Breast Cancer	46% Reduction in ORR	35% Reduction	7% Reduction	Level 6	Only licensed in combination with nab-paclitaxel for unresectable locally advanced or metastatic triple-negative breast cancer whose tumours have PD-L1 expression ≥1% and who have not received prior chemotherapy	• [TA639] Atezolizumab with nab paclitaxel is recommended for treating triple-negative, unresectable, locally advanced or metastatic breast cancer in adults whose tumours express PD L1 at a level of 1% or more and who have not had previous chemotherapy (no approval)
**Cetuximab or Panitumumab** (Colorectal Cancer)	Allow intermittent treatment with chemotherapy regimens that contain cetuximab or panitumumab	1	**COIN-B**: Intermittent chemotherapy plus either intermittent or continuous cetuximab for first-line treatment of patients with KRAS wild-type advanced colorectal cancer:	5.4-month Reduction in OS	7% Reduction	4% Reduction	Level 3	Treatment of epidermal growth factor receptor expressing, RAS wild-type metastatic colorectal cancer in combination with irinotecan-based chemotherapy, or first-line in combination with FOLFOX, or as a single agent in patients who have failed oxaliplatin- and irinotecan-based therapy and who are intolerant to irinotecan	• [TA439] Cetuximab is recommended as an option for previously untreated epidermal growth factor receptor expressing, RAS wild-type metastatic colorectal cancer in adults in combination with FOLFOX or FOLFIRI (full approval)
**Nivolumab** (Colorectal Cancer)	Nivolumab instead of chemotherapy for the treatment of metastatic colorectal cancer with high levels of micro-satellite instability and/or deficient mis-match repair	1	**Checkmate 142**: Nivolumab in patients with metastatic DNA mismatch repair-deficient or microsatellite instability-high colorectal cancer:	N/A (DOR NR (2-month OS 73%))	N/A (Incidence of 55% reported)	NR	Level 3	Only licensed in combination with ipilimumab for the treatment of mismatch repair deficient or microsatellite instability-high metastatic colorectal cancer after prior fluoropyrimidine-based combination chemotherapy	• [TA716] Nivolumab with ipilimumab for previously treated metastatic colorectal cancer with high microsatellite instability or mismatch repair deficiency; alternatively, **pembrolizumab** [TA709] is recommended in this indication (no approval)
**Encorafenib** and **cetuximab** (Colorectal Cancer)	Encorafenib and cetuximab for BRAF positive metastatic disease instead of chemotherapy	1	**BEACON CRC**: Encorafenib, Binimetinib, and Cetuximab in BRAF V600E–Mutated Colorectal Cancer (doublet regimen)	2.8 month gain in PFS	11% Reduction	NR	Level 6	In combination with cetuximab, for the treatment of adult patients with metastatic colorectal cancer (CRC) with a BRAF V600E mutation, who have received prior systemic therapy	• [TA668] Encorafenib plus cetuximab is recommended, within its marketing authorisation, as an option for treating BRAF V600E mutation-positive metastatic colorectal cancer in adults who have had previous systemic treatment (partial approval)
**Nivolumab** (Endometrial Cancer)	Nivolumab instead of chemotherapy for microsatellite instability-high tumours	1	**NCI-Match** (Arm Z1D): Nivolumab in patients with MMR-deficient, non-colorectal cancer (13 out of 42 patients with endometrial cancer)	N/A (ORR -36%; Median OS 17.3 months^b^)	N/A (7% Grade 4 toxicity reported^b^)	NR^b^	Level 4	Not licensed in endometrial cancer	• Not Recommended; (Alternatively, **dostarlimab** [TA779] recommended for previously treated advanced or recurrent endometrial cancer with high microsatellite instability or mismatch repair deficiency) (no approval)
**Pembrolizumab** (Gestational or placental site trophoblastic tumour)	Pembrolizumab first-line or subsequent line instead of combination chemotherapy (change of sequence)	1 + 3	No evidence in first-line; Case series available in refractory settings	N/A	N/A	N/A	N/A	Not licensed in gestational or placental site trophoblastic tumour	• Commissioned (NHS England Reference 170027P) as third line treatment for patients assessed as high risk (no approval)
**Pembrolizumab** (Head and Neck Cancer)	Pembrolizumab as first-line immunotherapy instead of chemotherapy	1 + 2	**KEYNOTE-048:** Pembrolizumab alone or with chemotherapy versus cetuximab with chemotherapy for recurrent or metastatic squamous cell carcinoma of the head and neck	Non-inferior across total population (OS-14.9 months; benefit in CPS >1 and > 20)	28% reduction	20% reduction	Level 4	Monotherapy indicated for the first-line treatment in tumours express PD-L1 with a combined positive score ≥1	• [TA661] Pembrolizumab is recommended as an option for untreated metastatic or unresectable recurrent head and neck squamous cell carcinoma in adults whose tumours express PD-L1 with a CPS ≥1 (full approval)
**Pembrolizumab** (Non-Small Cell Lung Cancer)	Stop maintenance pemetrexed in combination with pembrolizumab	1	**KEYNOTE-189**: No direct comparison of maintenance treatment arms available; Toxicity compared with **KEYNOTE-024**	N/A	41% Reduction	15.6% reduction	N/A	Not licensed for maintenance treatment without pemetrexed	• [TA683] Pembrolizumab with pemetrexed and platinum chemotherapy is recommended for untreated, metastatic, non-squamous non-small-cell lung cancer with no driver mutation (no approval)
**Pembrolizumab** (Non-Small Cell Lung Cancer)	Allow pembrolizumab single agent as a first-line treatment for squamous or non-squamous non-small cell lung cancer and a PDL-1 score ≤50%	1	KEYNOTE-024: Pembrolizumab versus chemotherapy for previously untreated, PD-L1-expressing, locally advanced or metastatic non-small-cell lung cancer	4.6 month gain in OS^c^	23% Reduction	6% Reduction	Level 4	**Two Relevant licensed indications:** **A)**Pembrolizumab monotherapy indicated for first-line treatment of metastatic NSCLC in tumours express PD-L1 with a ≥50% (TPS) with no driver mutation **B)**Pembrolizumab monotherapy is indicated for the treatment of metastatic NSCLC in adults whose tumours express PD-L1 with a ≥1% TPS and who have received at least one prior chemotherapy regimen	• [TA531] Pembrolizumab is recommended as an option for untreated PD-L1-positive metastatic non-small-cell lung cancer (NSCLC) in adults whose tumours express PD-L1 > 50% TPS with no driver mutation (no approval)
**Osimertinib** (Non-Small Cell Lung Cancer)	Osimertinib as first-line therapy to delay the need for subsequent chemotherapy	1 + 2	No direct comparison available (results from FLAURA study reported); Toxicity compared from AURA-3^l^	8.7 month gain in PFS (PFS- 18.4 months; ORR-80%)	24% Reduction	11% Reduction	Level 3	Licensed for first-line treatment of adult patients with locally advanced or metastatic NSCLC with activating EGFR mutation	• [TA654] Osimertinib is recommended, within its marketing authorisation, as an option for untreated locally advanced or metastatic EGFR mutation-positive non-small-cell lung cancer (full approval)
**Durvalumab** (Non-Small Cell Lung Cancer)	Allow durvalumab be given 4-weekly in patients eligible for durvalumab following treatment with chemo-radiotherapy	3	Based on several trials (e.g., PACIFIC, CASPIAN trials); Real-world pooled data used for comparison	No difference	1.2% Reductions	NR	N/A	Dose recommended (10 mg/kg every 2 weeks or 1500 mg every 4 weeks)	• [TA798] Durvalumab is recommended as an option for treating locally advanced unresectable non-small-cell lung cancer (NSCLC) in adults whose tumours express PD-L1 ≥1% and whose disease has not progressed after platinum-based chemoradiation (full approval)
**Carboplatin and paclitaxel** (Non-Small Cell Lung Cancer)	Switch to carboplatin and paclitaxel from day 8 treatments such as gemcitabine and carboplatin and cisplatin and vinblastine	3	NVALT-3: Quality of life, geriatric assessment and survival in elderly patients with NSCLC treated with carboplatin–gemcitabine or carboplatin–paclitaxel	8% Reduction in ORR; 1.7 month reduction in OS	15% Reduction	9% Reduction	Level 6	All drugs approved in this setting	• Both available unrestricted
		3	No comparator for cisplatin and vinblastine	N/A	N/A	N/A	Level 6		
**Dabrafenib plus Trametinib** (Non-Small Cell Lung Cancer)	Dabrafenib plus trametinib for BRAF positive metastatic disease instead of chemotherapy	1 + 2	Study BRF113928: Dabrafenib plus trametinib in patients with previously untreated BRAF V600E mutant metastatic non-small-cell lung cancer (single arm, no comparator available)	N/A (Reported ORR 64%; DOR-14.6 months)	N/A (Rate = 70%)	N/A (Rate = 0%)	Level 3	Dabrafenib in combination with trametinib is indicated for the treatment of adult patients with advanced non-small cell lung cancer with a BRAF V600 mutation	• [TA898] Dabrafenib plus trametinib is recommended as an option for treating BRAF V600 mutation-positive advanced non-small-cell lung cancer (both full approval)
		1 + 2	Study BRF113928: Dabrafenib plus trametinib in patients with previously treated BRAF V600E mutant metastatic non-small-cell lung cancer (single arm; no comparator available)	N/A (Reported ORR 63%; PFS-9.7 months)	N/A (Rate = 49%)	N/A (Rate = 0%)	Level 5		
**Chemotherapy**^d^ (Small Cell Lung Cancer)	Stop first-line chemotherapy^d^ for stage 4 small cell lung cancer after 4 cycles	1 + 3	Duration of Chemotherapy for Small Cell Lung Cancer: A Meta-Analysis	No difference in OS/PFS (some PFS benefit to maintenance chemotherapy in extensive stage disease)	NR		N/A	Licensed in this indication	• Available unrestricted
**Oral Therapy**^e^ (Melanoma)	Give oral therapy^e^ as first-line treatment for BRAF-positive patients in preference to immunotherapy	2 + 3	Outcome of melanoma patients with elevated LDH treated with first-line targeted therapy or PD-1-based immune checkpoint inhibition	30% reduction in PFS at 12-months compared to anti-PD-1/CTLA-4 combination; 4% improvement in PFS at 12-months compared to anti-PD-1 therapy alone	NR		N/A	Multiple BRAF and MEK (including dabrafenib and trametinib) approved	• [TA396] Trametinib in combination with dabrafenib for treating unresectable or metastatic melanoma (full approval)
**Immunotherapy Doublet** (Melanoma)	Stop immunotherapy doublet (ipilimumab and nivolumab) and switch to single agent nivolumab or pembrolizumab	3	CheckMate 067: Study of Nivolumab or Nivolumab Plus Ipilimumab Versus Ipilimumab Alone in Previously Untreated Advanced Melanoma (no matched study of pembrolizumab)	23.1^f^ month reduction in OS; 4.6 month reduction in PFS	36% Reduction	NR^n^	Level 3	Licensed as monotherapy in this indication	• [TA384] Nivolumab as monotherapy is recommended for treating advanced (unresectable or metastatic) melanoma in adult • [TA366] Pembrolizumab is recommended for treating advanced (unresectable or metastatic) melanoma that has not been previously treated with ipilimumab (full approval)
**Nivolumab** (Mesothelioma)	Nivolumab monotherapy instead of second line chemotherapy.	1	CONFIRM: Nivolumab versus placebo in patients with relapsed malignant mesothelioma (no comparator as compared to placebo)	N/A (Reported ORR 11%; PFS 3 months; OS 10.2 months)	N/A (Rate = 40%)	N/A (Rate = 0.5%)	Level 6	Monotherapy not licensed	• Available on the CDF [NIV13CV_v1.1]
**Temozolomide plus capecitabine** (Neuroendocrine Tumours)	Give oral temozolomide and capecitabine instead of intravenous streptozocin and 5-fluorouracil	2	Comparison of Temozolomide-Capecitabine to 5-Fluorouracil-Dacarbazine^o^ in 247 Patients with Advanced Digestive Neuroendocrine Tumors Using Propensity Score Analyses (no direct comparison available)	3.5% increase in ORR; 4.4 month gain in PFS (global)	16.2% increase	2.2% increase	Level 4	Licensed in this indication	• Available unrestricted
**Olaparib, niraparib or rucaparib** (Ovarian Cancer)	Give olaparib, niraparib or rucaparib (poly-ADP-ribose [PARP] inhibitors) instead of chemotherapy plus maintenance PARP inhibitor at first relapse for BRCA-positive PARP-naive patients	1 + 2	No direct comparison available; Toxicity^g^ compared from ARIEL4	N/A	21% increase	5% decrease	N/A	Not Licensed	• Not approved (no approval)
**Trametinib** (Ovarian Cancer)	Trametinib for advanced low grade serous ovarian carcinoma	1 + 2	GOG281/LOGS: Trametinib versus standard of care in patients with recurrent low-grade serous ovarian cancer	5.6 month increase in PFS	NR	2% reduction	Level 4	Not licensed	• Available on the CDF [TRAM1CV]
**Enzalutamide** with **androgen deprivation therapy** (Prostate Cancer)	Enzalutamide with androgen deprivation therapy for patients with newly diagnosed metastatic disease instead of docetaxel	1 + 2 + 3	No direct comparison available; toxicity taken from ENZAMET study^h^	N/A	91% reduction^h^	5% reduction	N/A	Licensed in this indication	• [TA712] Enzalutamide plus androgen deprivation therapy (ADT) is recommended, within its marketing authorisation, as an option for treating hormone-sensitive metastatic prostate cancer in adults (full approval)
**Abiraterone** (Prostate Cancer)	For patients who are intolerant of enzalutamide, give the option of switching treatment to abiraterone^i^	1 + 2 + 3	STAMPEDE: Adding abiraterone or docetaxel to long-term hormone therapy for prostate cancer	No difference in OS or prostate-cancer specific survival	2% reduction	12% reduction	Level 3	Licensed in both hormone-sensitive and castrate resistant prostate cancer (not specific to indication)	• Approved in the metastatic hormone-relapsed [TA387] and castration-resistant previously treated with docetaxel [TA259] but not treating newly diagnosed high-risk hormone-sensitive metastatic prostate cancer [TA721] (full approval)
**Nivolumab or oral therapy** (Renal Cell Cancer)	Stop first-line immunotherapy using nivolumab with ipilimumab in intermediate and poor risk groups, and switch to either first-line single agent nivolumab or use oral therapy as first-line and nivolumab with ipilimumab as second-line therapies	2 + 3	Nivolumab monotherapy first-line: **HCRN GU16-260-Cohort B** study of nivolumab and salvage nivolumab/ipilimumab in treatment-naïve patients with advanced non-clear cell renal cell carcinoma	N/A (ORR-14.3%; PFS- 4 months)	N/A (20% rate reported)	N/A (nil reported)	Level 6	Not licensed	• Not approved (no approval)
		2	Oral therapy (sunitinib) as first line: CheckMate 214 - Nivolumab plus ipilimumab versus sunitinib in first-line treatment for advanced renal cell carcinoma	15% reduction in ORR; 3.2 month lower PFS; 8% lower 12-month overall survival	17% increase	NR^n^ (either arm)	Level 6	Licensed (also other drugs in this setting (e.g., pazopanib))	• [TA169] Sunitinib is recommended as a first-line treatment option (full approval)
		3	Nivolumab/Ipilimumab 2nd line: FRACTION-RCC - nivolumab plus ipilimumab for advanced renal cell carcinoma after progression on immuno-oncology therapy (no comparator)	N/A (ORR-17%; PFS-3.7 months; OS-23.8 months)	N/A (28.3% rate reported)	N/A (nil reported)	Level 6	Not licensed	• Not approved (no approval)
**Nivolumab** or **oral therapy** (Renal Cell Cancer)	Use first- and second-line oral tyrosine kinase inhibitors and switch nivolumab from second to third line	2 + 3	CheckMate 025 Nivolumab versus Everolimus in Advanced Renal-Cell Carcinoma (3rd line patients)	4% improvement in ORR (2nd versus 3rd)	2% reduction	NR^n^	Level 4	Licensed for advanced renal cell carcinoma after prior therapy	• [TA417] Nivolumab is recommended, within its marketing authorisation, as an option for previously treated advanced renal cell carcinoma in adults (no approval)
**Nivolumab** (Upper gastrointestinal cancers including oesophageal, gastric, small bowel, biliary tract & pancreatic)	Nivolumab instead of chemotherapy for microsatellite instability-high tumours (MSI-H)	1	No direct comparison available: Toxicity compared from ATTRACTION-3 (only applicable to oesophagus^j^), no applicable studies for gastric, small bowel, biliary tract or pancreatic identified	N/A	7% reduction	28% reduction	N/A	Not licensed^k^	• Available on the CDF [NIV12CV]

**OS**, Overall Survival; **DFS**, Disease Free Survival; **pCR**, Pathological Complete Response; **PFS**, Progression Free Survival; **ORR**, Overall Response Rate; **DOR**, Duration of Response; **NR**, Not Reported; N/A, Non-Assessable.

Other Abbreviations: **PDL1**, Programmed death-ligand 1; **HER2**, Human epidermal growth factor receptor 2; **CDF**, Cancer Drug Fund; **FOLFOX**, Chemotherapy regimen made up of the drugs folinic acid, fluorouracil, and oxaliplatin; **FOLFIRI**, Chemotherapy regimen made up of the drugs folinic acid, fluorouracil, and irinotecan; **MMR**, Mismatch Repair; **TPS**, tumour proportion score; **EGFR**, Epidermal growth factor receptor.

a All breast cancer types (e.g., Luminal A/B etc).

b Results reflect all non-colorectal cancer and not specific to endometrial cancer.

c Value reflects comparison in the TPS >1% group (Pembrolizumab versus Chemo-alone).

d Platinum-based and Etoposide therapy (NCCN grade 1 classification).

e Combination of BRAF/MEK (e.g., dabrafenib/trametinib deemed most appropriate (NCCN grade 1 classification)).

f In Checkmate-067, the OS in the nivolumab/ipilimumab has not yet been reached but is estimated >60 months, so a value of 60-month has been used in this calculation.

g ARIEL-4 selected for comparison (direct comparison of rucaparib versus chemotherapy in selected patient group (included both platinum sensitive and resistant groups)).

h ENZAMET study used for toxicity (Anti-androgen + docetaxel compared to Ani-androgen + Enzalutamide arms compared; Selected toxicities (as reported) only).

i Assumed instead of docetaxel (guidelines however not explicit about setting or replacement therapy).

j ATTRACTION-3 study is a study of Nivolumab versus chemotherapy in patients with advanced oesophageal squamous cell carcinoma refractory or intolerant to previous chemotherapy and is not specific to MSI-H.

k Pembrolizumab does hold a UK license for treatment of the following MSI-H (or dMMR) tumours in adults with unresectable or metastatic gastric, small intestine, or biliary cancer, who have disease progression on or following at least one prior therapy.

l Direct comparison of chemotherapy versus Osimertinib used for toxicity analysis rather than comparative studies of tyrosine kinase inhibitors (e.g., FLAURA study) due to greater relevance.

m Date of censoring is 1st January 2024.

n Only Adverse Events with incidence >10% reported.

o Similar mechanism of action to streptozocin.

p All therapies deemed to meet criteria 4 & 5 for inclusion (not therefore assessed).

In this study we sought to identify new oncology therapies approved for solid cancers, recommended during the COVID-19 pandemic by NHS England as interim treatment options (NICE guidelines [NG161]); (Appendix). We evaluated published evidence (e.g., pivotal trials); supporting interim therapy selection (comparing to baseline treatments), mapping clinical outcomes to the selection criteria (Table 2) and then categorising these based on treatment intent and risk-benefit ratio (Table 1). We evaluated the potential benefit these may offer in terms of severe adverse events, due to a higher probability of requiring medical intervention or hospital admission, and severe neutropenia, resulting in increased risk of sequelae from SARS-CoV-2 infection. Finally we established the availability of these interim therapy options at the end of the pandemic.

## Methods

We evaluated new interim SACT therapy options endorsed for solid cancer by NHS England from the start of the COVID-19 pandemic (December 2019) until the withdrawal of interim COVID-19 therapy options (August 2022). Any therapy for solid cancer included on this list during the study period (December 2019–August 2022) where included in this analysis. This cohort study followed the STROBE reporting guidelines. This study was not submitted for institutional review board review because it used publicly available data and was not considered human subject research.

### Data sources

We performed a retrospective analysis using data extracted from NICE guideline [NG161] COVID-19 rapid guideline: delivery of SACT.^10^ This guideline lists new interim oncology treatment options (Appendix), first published in March 2020. The interim list was updated quarterly, with options added and removed based on clinical evidence, or becoming available through standard NICE commissioning. The last version of interim options was accessed in August 2022. Notably, these are no longer accessible on the NICE website, being superseded by post-pandemic guidance.

All available data was extracted from COVID-19 interim treatment options to categorise therapy, tumour site, treatment setting (e.g., neoadjuvant, adjuvant, metastatic), specific indication, suggested replacement/baseline therapy option and rationale for inclusion if specified (e.g., reduced immunosuppression). Many interim options contained multiple indications (multi-faceted), for example nivolumab monotherapy in renal cell cancer could be used as first- or third-line therapy, and were therefore considered as separate options for analysis. For each therapy identified (including multi-faceted indications) the Electronic Medicines Compendium (EMC) website (https://www.medicines.org.uk/emc), which contains a database of all medicines licensed for use in the UK, and has been checked and approved by either the UK (e.g., MHRA) or European government agencies responsible, was evaluated to identify if this therapy held a valid UK market authorisation or was ‘off-label’ (date of censoring January 2024).^21^ Therapies were deemed to be fully licensed if the complete indication was specified on the EMC webpage. If only aspects of the interim indication were covered, then the drug was deemed to be partially covered. For example, atezolizumab in bladder cancer permitted first-line use across all patient types, however UK market authorisation specifies only use for patients considered cisplatin ineligible and tumours with PDL1 expression ≥5%, thus covering only part of the interim indication. If UK market authorisation did not apply to any part of the interim indication, then the use was considered ‘off label’. The EMC webpage was analysed at the time of specification of new interim indications, and again at the date of censoring to ensure all changes were accounted for.

For interim COVID-19 therapy indications which held UK market authorisation, the pivotal trials used for regulatory approval were utilised for efficacy and safety analysis. If interim therapies were not licensed in the interim indication (e.g., ‘off-label’ use) other regulatory databases, such as the European Medicines Agency (EMA) or US Food and Drug Administration (FDA), were searched to identify appropriate clinical trials for analysis. If no appropriate studies could be identified then a PubMed search (see Appendix for Search terms and all trials used for analysis) was performed to identify other appropriate randomised controlled trials, meta-analysis, real world studies and cohort studies, to allow evaluation of comparative efficacy and/or safety. Finally for each interim therapy indication the NICE website and national Cancer Drugs Fund list (https://www.england.nhs.uk/cancer/cdf/cancer-drugs-fund-list/) was cross-checked to identify if interim therapies had undergone formal approval (commissioning) by NHS England or remained available via the Cancer Drugs Fund.

### Data analysis

Comparative clinical trials were analysed to identify appropriate clinical outcomes. In the metastatic setting, overall survival (OS) and, if not available surrogate endpoints progression free survival (PFS) and overall response rate (ORR) were selected, allowing assessment against COVID-19 prioritisation guidelines (Table 1), and for neoadjuvant and adjuvant options, pathological complete response (pCR) and disease-free survival (DFS) were used respectively. If these were not reported, or not comparable between the baseline therapy and new interim therapy, then clinical studies comparing toxicity were selected in a setting deemed closest to the original study to permit some analysis. For example, nivolumab was recommended as an interim therapy option for microsatellite instability-high upper gastrointestinal cancers (e.g., oesophageal) instead of chemotherapy, however, efficacy has not been directly compared, but safety information can be gleaned from the ATTRACTION-3 study.

Comparative efficacy was extracted by comparing clinical outcomes (e.g., ORR and OS) between the interim therapy and the treatment they replaced (baseline therapy). If these were compared directly in clinical trials (e.g., different treatment arms) these values were used. If there were no clinical trials directly comparing efficacy outcomes directly, then clinical benefit from equivocal studies (e.g., meta-analysis, retrospective cohorts), using the same clinical outcomes e.g., OS, PFS or ORR) were compared.^8^ A similar methodology was utilised to assess the safety profile using the National Cancer Institute Common Terminology Criteria for Adverse Events (v4.0). However, if there were no clinical trials directly comparing safety outcomes, then safety data from equivocal studies was evaluated. Incidences of grade 3 and 4 toxicity, and specifically neutropenia (significant risk factor for sequelae from SARS-CoV-2 infection) were compared between interim therapies and baseline treatment(s).^16^ This allowed interim therapies to be evaluated against COVID-19 Selection Criteria for Interim Treatment Options (Table 2).

NHS England interim COVID-19 SACT recommendations were withdrawn in August 2022, we therefore sought to evaluate which interim therapies remained available. All interim therapies were compared with current NICE guidelines to establish availability at the date of censoring. Therapy indications were deemed to be fully available if covered entirely by the NICE recommendation and partially if only aspects were covered.

### Role of the funding source

No funding was obtained for this study.

## Results

In total 31 indications were included as COVID-19 interim therapies options for 14 different solid cancers. 4 indications were multi-faceted, i.e. containing ≥2 indications (e.g., nivolumab in renal cell cancer could be used as first line (instead of nivolumab/ipilimumab) or 3rd line (after tyrosine kinase therapy) treatment), meaning a total of 36 interim therapy options were available for analysis (Table 3). Interim therapy options were focused (83%; 30/36) on treatment modification(s) (alteration of the standard treatment) in the metastatic setting, however adjuvant (14%; 5/36) and neoadjuvant (3%; 1/36) therapy options were also available. The cancer types with the highest number of interim options were lung (22%; 8/36), breast (19% 7/36) and colorectal cancer (8% 3/36). The majority focused on immune checkpoint inhibitors (39%; 14/36) and targeted therapies (33% 12/36). Interim treatments frequently recommended switching from intravenous to oral SACT (31%; 11/36) (Table 2). The majority (81%; 25/31) specified rationale for inclusion as interim therapies, including reduced immunosuppression (16%; 5/31), neutropenia (10%; 3/31), lower toxicity (13%; 4/31) and reduced hospital visits/stays (13%; 4/31).

### Comparative efficacy

The comparative efficacy of interim COVID-19 treatment options was assessable for 23 (64%; 23/36) therapies, using sixteen randomised controlled trials, directly comparing efficacy of interim options compared to baseline therapies, three meta-analyses, two retrospective cohort studies, one non-randomised phase two trial and one phase one trial. For 13 (36%; 13/36) interim therapies no appropriate comparator study comparing interim option with replacement therapy could be identified, precluding analysis. Of the 23 interim options which could be assessed, 9 (39%; 9/23) had superior efficacy, compared to baseline therapies, with 45% (4/9) having superior ORR, 33% (3/9) PFS and 22% (2/9) OS. 8 (35%; 8/23) interim therapies had lower efficacy than comparator, including lower ORR (37%; 3/8), PFS (25%; 2/8), OS (25%; 2/8) and pCR (12%; 1/8), 4 (17%; 4/23) interim therapies showed equivocal efficacy, and a further 2 (9%; 2/23) demonstrated non-inferiority.

### Comparative toxicity and neutropenia

The comparative toxicity of interim treatment options compared to baseline options was assessable for 22 (61%; 22/36) therapies using 17 randomised controlled trials, directly comparing efficacy of interim options compared to baseline therapies, two retrospective cohort studies, one phase one trial and for two interim treatments, two different randomised controlled trials in the same setting where compared. Overall, 18 (81%; 18/22) interim therapies demonstrated a lower reported incidence of grade 3 and 4 toxicities and 1 (4%; 1/22) equivocal toxicity compared to baseline treatments. 3 (14%; 3/22) interim therapies had a higher incidence of grade 3 and 4 toxicity. Comparable toxicities were not assessable for 12 (39%; 12/36) interim options due to no appropriate comparator study being identified.

Comparable incidences of significant neutropenia (grade 3 or 4) between interim therapies and baseline treatments were available for 17 (47%; 17/36) therapies using 15 randomised controlled trials, directly comparing efficacy of interim options compared to baseline therapies, one retrospective cohort study, one phase one trial and for two interim treatments, two different randomised controlled trials in the same setting were compared. 16 (94%; 16/19) interim therapies offered a lower incidence of significant neutropenia compared to baseline treatments. Only 1 (6%; 1/17) interim therapy, temozolomide plus capecitabine in neuroendocrine cancer, showed an increased incidence of neutropenia compared to baseline therapy. Comparative neutropenia incidence was not assessable for 19 (53%; 19/36) interim therapies.

### Treatment prioritisation and COVID 19 selection criteria

**Fig. 1:**
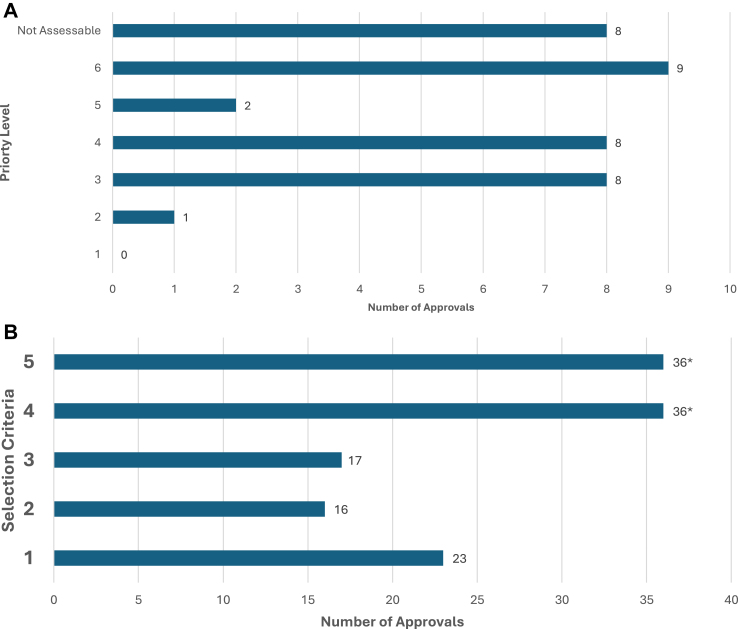

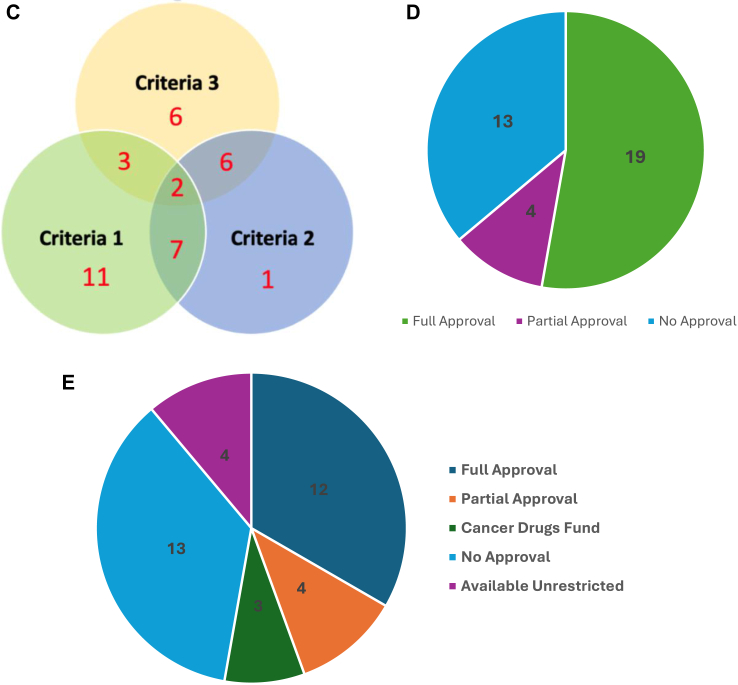
**A:** Categorisation of Systemic Anti-Cancer Therapies (SACT) based on treatment intent and risk-benefit ratio. Treatments were classified based on NHS COVID-19 prioritisation guidance [NG161] (Table 1). **B:** Number of therapies meeting each Selection Criteria for Interim Treatment Options During the COVID-19 Pandemic. ∗All therapies deemed to meet criteria 4 and 5 for inclusion. Treatments were classified based on NHS COVID-19 prioritisation guidance [NG161] (Table 2). **C:** Number of therapies meeting each selection criteria for inclusion as an interim treatment options. Treatments were classified based on NHS COVID-19 prioritisation guidance [NG161] (Table 2). **D**: UK Market Authorisation of Interim Treatment Options Approved During the COVID-19 Pandemic. **E**: Availability of Interim Treatment Options at the End of the COVID-19 Pandemic.

Utilising NHS COVID-19 prioritisation guidance (Table 1) we were able to categorise 28 (77%; 28/36) interim therapies. The remaining eight (23%; 8/36) could not be classified due to uncertain clinical efficacy evidence (e.g., pembrolizumab in gestational trophoblastic disease). For interim therapies (Fig. 1A) which could be classified, no therapy option achieved the highest ranking (Priority 1), one (4%; 1/28) treatment was classified as priority 2 and eight (29%; 8/28) as priority 3. The majority, 19 (67%; 19/28) interim therapies, achieved a lower priority ranking (level 4–6).

Analysing interim therapy options using COVID-19 selection criteria (Table 2), all treatments were deemed to meet specification 4 (feasible to deliver) and 5 (adequate capacity), being mandatory for inclusion as options (Fig. 1B). All (100%; 36/36) interim therapies were deemed to meet ≥1 additional selection criterion. 18 (50%; 18/36) interim therapies met only one further selection criteria, including 11 (31%; 11/36) specification 1 (reduced immunosuppression), 1 (3%; 1/36) specification 2 (home administration) and 6 (16%; 6/36) specification 3 (less resources). 18 therapies (50%; 18/36) met ≥2 specifications (Fig. 1C).

Of the 25 therapies which specified an inclusion reason for inclusion (Appendix), we were able to verify 64% (16/25) interim therapies met the stated reason(s). For the remaining 9 (36%; 9/25) therapies no comparative study could be identified to validate inclusion reason and remain unvalidated.

### UK market authorisation and access

19 (53%; 19/36) interim therapies held full UK market authorisation (Fig. 1D). A further four (11%; 4/36) therapies were deemed to hold partial market authorisation, covering aspects of recommended usage. 13 (36%; 13/36) interim therapies options did not hold a full/partial UK market authorisation and were considered ‘off-label’ in the interim indication.

Analysing access to interim options at the end of the pandemic (May 2023) identified 19 (53% 19/36) interim options were fully available, and a further four (11% 4/36) therapies were partially available (Fig. 1E). Of the 19 interim options fully available, 12 (35%; 12/36) are NICE recommended (allowing access by NHS England), 3 (8% 3/36) are available via the Cancer Drugs Fund as interim therapy options, and a further 4 (11%; 4/36) are available unrestricted (all cytotoxic chemotherapy). For the 12 NICE recommended therapies, six (50%; 6/12) were newly recommended (e.g., Osimertinib as first-line therapy in NSCLC) following the cessation of NICE COVID guidelines [NG161], of which the majority (66% 4/6) showed improved or equivalent efficacy. Six (50%) of the 12 were already NICE approved pre-pandemic but had been superseded by more efficacious options but due to other pandemic considerations (e.g., toxicity) were recommended in place of existing treatments, for example NICE interim therapy guidance recommended immunotherapy monotherapy (e.g., pembrolizumab) in place of doublet therapy (e.g., nivolumab/ipilimumab) for metastatic melanoma. For the 13 (36% 13/36) interim therapies no longer available, 23% (3/13) had superior efficacy compared to baseline options, suggesting other factors (e.g., price, commercial factors) may account for their withdrawal. A further 23% (3/13) had worse efficacy compared to baseline therapies, and the majority (54% 7/13) had no comparable efficacy evidence to support usage.

## Discussion

In this study we analysed the clinical evidence supporting interim SACT options during the COVID-19 pandemic, demonstrating that comparative evidence against replacement (baseline) treatments was only available for 64% (23/36) of interim therapies. For interim therapy (36%; 13/36) options where no comparator or no direct reference could be identified (e.g., PARP inhibitors, instead of chemotherapy, at first relapse for BRCA-positive PARP-naive patients ovarian cancer patients) it remains unknown what evidence, beyond the clinical opinion of the Chemotherapy Clinical Reference Group (CCRG), justified inclusion. Furthermore, it is also unknown if any CCRG members may have held any relevant conflicts of interest. 47% (17/36) of interim therapies did not hold a full UK Market Authorisation meaning prescribing would be considered ‘off label’, placing greater responsibility upon the prescriber, this was reinforced by interim guidance stating, “responsibility for using these interim treatment regimens lies entirely with the prescribing clinician, who must discuss the risks and benefits of interim treatment regimens with individual patients, their families and carers” (Appendix).^22^

The paucity of data supporting these interim options would have represented a significant challenge for prescribers. As Interim treatment regimens were selected based on clinical opinion(s) from members of the CCRG, and being endorsed by NHS England and NHS Improvement, clearer communication of the rationale for inclusion, in terms of both efficacy and safety, and the proposed use, would have been extremely valuable to prescribers providing informed consent, and concordance of interim SACT options. Furthermore, as 35% of interim therapies involved switching to less efficacious options, the understanding, and quantification, in terms of other proposed benefits (e.g., reduced immunosuppression) related to the pandemic would have been important to communicate. This represents a missed opportunity to better inform prescribers of the rationale behind interim treatment selections. In terms of future pandemic planning, it is important to ensure clearer, and more robust, frameworks are in place to inform prescribers, and other healthcare professionals, of the evidence supporting these recommendations.

The COVID-19 rapid NICE guidelines for delivery of SACT were introduced to maximise cancer patient safety and facilitate the more rationale use of constrained NHS resources, throughout the pandemic. New SACT registrations fell sharply at the start of the pandemic, likely due to concerns around SACT safety (e.g., cytotoxic chemotherapy).^23^ However, following the introduction of COVID-19 interim treatment options new SACT registrations rebounded sharply, even surpassing pre-pandemic monthly levels, demonstrating a high, and accelerated, uptake of these new interim treatment options, especially for some drugs (e.g., enzalutamide in prostate cancer).^23^ Notably, a similar pattern was also seen in other UK countries (e.g., Scotland), who introduced their own equivalent SACT guidance.^24^^,^^25^ Further, it is perhaps unsurprising that half (16/36) of interim therapy options focused on three out of the four commonest cancers (lung, breast and colorectal) in the UK, likely associated with considerable resource-saving (e.g., chemotherapy chair time, pharmacy formulation time, monitoring requirements etc), but higher costs for the NHS.

For all interim therapy options, we were able to identify at least one COVID-19 selection criteria (in addition to mandatory criteria) to warrant inclusion in this guidance, with 44% (16/36) meeting ≥2 criteria concomitantly. However, assessing interim treatment options, based on treatment intent and risk-benefit ratio (Table 1) for treatment prioritisation demonstrated most therapies would achieve only a low priority (Priority 4–6) for clinical delivery, questioning the importance and true benefit these interim therapies offered. This is partly due to a smaller number (17%; 6/36) of non-metastatic (e.g., adjuvant, neoadjuvant) interim treatment options being included which typically receive higher priority ranking (Priority 1–4) compared to metastatic/non-curative treatment options (Priority 3–6), however it does reflect the uncertainty or lower efficacy of many metastatic treatment options. Furthermore, this is reflected in current NICE recommendations, with interim COVID-19 treatment options which received a higher priority (1–3) more likely to be available at the end of the pandemic compared to those which achieved a lower priority (4–6) ranking. Many low priority (4–6) ranked treatment options have been withdrawn, suggestive of little clinical value and/or unsustainable costs.

The administration of cytotoxic chemotherapy, and its concomitant immunosuppressive effects, was linked to deleterious outcomes in cancer patients who contracted SARS-CoV-2 in large UK retrospective studies, particularly those with baseline neutropenia.^26^ Therefore, it is unsurprising that the majority (52%) of interim therapy options advocated switching of cytotoxic chemotherapy to less immunosuppressive therapies (e.g., immune checkpoint inhibitors), potentially safeguarding cancer patients against serious sequelae from SARS-CoV-2 infection. Paradoxically, some interim treatment options had increased rates of neutropenia (e.g., temozolomide and capecitabine) or severe toxicities (e.g., sunitinib) compared to baseline options. However, in each case interim treatments offered other benefits (e.g., oral administration) (Table 2), potentially showing the balance given to each of the COVID-19 prioritisation criteria. Publication of the evidence supporting the recommendation of these interim options, would have enabled greater understanding of the benefits, and risks, of each treatment relative to the COVID-19 selection criteria governing inclusion.

The clinical outcomes of patients receiving these options remains unknown, reflecting a missed opportunity to understand interim therapy efficacy in a real-world setting, beyond eligibility criteria for clinical trials, and to inform about the viability of this strategy for future pandemic planning. Furthermore, as many patients may have had significant risk factors (e.g., >75 years or significant co-morbidities) for severe COVID-19 sequalae, it is unknown what consequence treatment (versus no treatment) may have had. Rigorous data collection, and rapid publication, of clinical outcomes in cancer patients was highly successful in informing treatment and vaccination decisions during the pandemic through rapid establishment of cooperative working groups (e.g., CCC19, OnCOVID, TERAVOLT).^4^ A similar approach could have proven successful in assessing, and modifying, interim therapy options, and should be considered in future pandemic planning.

Our work reflects the need for significant thinking around the advisory and legislative framework for national interim ‘guidance’ for cancer, and beyond, in the face of any future global healthcare crisis, acknowledging that implementing any new healthcare guidance, in cancer and beyond, during any healthcare emergency could be challenging. New frameworks need to be highly versatile and adaptive, allowing for rapid modifications when presented with new data, and involve additional stakeholders (e.g., MHRA) to allow for rapid modifications in drug labels based on recommendations. Future pandemic preparedness and resilience requires not just ‘lessons learnt’ from previous national experiences but also forward planning with an ability to adapt rapidly when presented with new data.

This study has several limitations which merit discussion. Firstly, some interim options could not be compared to baseline therapies due to a lack of appropriate comparative studies (e.g., PARP inhibitors instead of chemotherapy in frontline ovarian cancer). Therefore, these could not be compared meaningfully, however where possible relevant safety data (e.g., ARIEL-4) was analysed to provide insight. Secondly for some interim indications, clinical outcomes were not compared directly in clinical trials (e.g., different controlled arms), so had to be inferred from closely related studies. Thirdly, the scope of this study was focused on interim therapies for solid cancers, however several therapies were also approved for haematological malignancies. These were not included due to the scope of expertise of the author team but would provide further valuable insight. Fourthly, when primary OS data from pivotal trials was not available, alternative surrogate outcomes (e.g., PFS, ORR, pCR etc) as recognised by medicines agencies (e.g., the Food and Drug Administration) were used instead for comparison. Despite being useful, surrogate endpoints may not correlate well with clinical outcomes in some settings (e.g., pCR in early breast cancer).^27^ Finally, some interim baseline options (e.g., cisplatin and vinblastine in non-small cell lung cancer) are not listed in international guidelines (e.g., ESMO, NCCN) used for analysis and may represent an inaccuracy in the original listing, precluding further evaluation.

### Conclusion

During the COVID-19 pandemic NHS England approved 31 interim therapies (across 36 indications) for solid cancers. The majority focused on greater use of immune checkpoint inhibitors and targeted treatments, in place of cytotoxic chemotherapy, conferring potential benefits in terms of reduced immunosuppression. For one-third of interim treatment options no objective clinical evidence could be identified to justify inclusion. Of those with evidence, 65% offered improved or equivocal clinical efficacy compared to baseline therapies, however around one-third of interim treatments had lower clinical benefit. Nearly all interim treatment options offered other benefits (e.g., reduced immunosuppression) in the context of the COVID-19 pandemic. Following the cessation of COVID-19 interim treatment guidelines and integration into routine care, only half of all treatment options remain fully available.

## Contributors

ML and AE performed the data collections, analysis and drafting of the manuscript. AA, RS, HN and JK provided critical review. ML, AA and RS responded to reviewers comments. All authors take responsibility for the data analysis.

## Data sharing statement

Only publicly available data was used in this analysis. All data used in this analysis can be accessed via the references or information in the appendix.

## Declaration of interests

ML, AE, RS, JK declare no conflicts of interest. HN reported receiving grants from the Commonwealth Fund, Health Foundation, UK Research and Innovation, and National Institute for Health and Care Research and personal fees from the World Health Organization, BMJ, and Pharmaceutical Group of the European Union outside the submitted work. AA is the recipient of an Advanced Fellowship NIHR300599 from the National Institute for Health Research. The views expressed in this publication are those of the author(s) and not necessarily those of the NHS, the National Institute for Health Research or the Department of Health and Social Care.
