# Supplementary material for: Efficacy and safety of interim oncology treatments introduced for solid cancers during the COVID-19 pandemic in England: a retrospective evidence-based analysis
Source: Lancet Reg Health Eur. 2024 Sep 10;46:101062. doi: 10.1016/j.lanepe.2024.101062 (PMC11415950; doi:10.1016/j.lanepe.2024.101062)
Supplement: Appendix [file mmc1.docx]

**Appendix**

**Search Terms:**

Pivotal trials and original trial data were searched from the PubMed database for each interim drug indication. Peer-reviewed articles and presentations of study results held on academic conferences were identified.

The search strategy included the cancer site, drug name, indication, if given, and search terms for comparators, if given, “toxicity”, “safety” and “quality of life”. The search was repeated for each drug indication and in case without EMA approval, study data were chosen that were most similar to the respective interim treatment option.

**Pivotal Trials Used for Analysis:**

Galsky MD, Arija JÁA, Bamias A, *et al.* Atezolizumab with or without chemotherapy in metastatic urothelial cancer (**IMvigor130**): a multicentre, randomised, placebo-controlled phase 3 trial. *The Lancet* 2020; **395**: 1547–57.

- Group B (atezolizumab monotherapy)
- Group C (platinum-based chemotherapy)

Coleman R, Gray R, Powles T, *et al.* Adjuvant bisphosphonate treatment in early breast cancer: Meta-analyses of individual patient data from randomised trials. *The Lancet* 2015; **386**: 1353–61.

Earl HM, Hiller L, Vallier AL, *et al.* 6 versus 12 months of adjuvant trastuzumab for HER2-positive early breast cancer (**PERSEPHONE**): 4-year disease-free survival results of a randomised phase 3 non-inferiority trial. *The Lancet* 2019; **393**: 2599–612.

Gianni L, Pienkowski T, Im YH, *et al.* Efficacy and safety of neoadjuvant pertuzumab and trastuzumab in women with locally advanced, inflammatory, or early HER2-positive breast cancer (**NeoSphere**): A randomised multicentre, open-label, phase 2 trial. *Lancet Oncol* 2012; **13**: 25–32.

Huober J, Weder P, Veyret C, *et al.* **PERNETTA**: A non-comparative randomized open label phase II trial of pertuzumab (P) + trastuzumab (T) with or without chemotherapy both followed by T-DM1 in case of progression, in patients with HER2-positive metastatic breast cancer (MBC): (SAKK 22/10 / UNICANCER UC-0140/1207). *Annals of Oncology* 2018; **29**: viii93.

Talbot DC, Moiseyenko V, Van Belle S, *et al.* Randomised, phase II trial comparing oral capecitabine (Xeloda®) with paclitaxel in patients with metastatic/advanced breast cancer pretreated with anthracyclines. *British Journal of Cancer 2002 86:9* 2002; **86**: 1367–72.

Gradishar WJ, Tjulandin S, Davidson N, *et al.* **CA012** Phase III trial of nanoparticle albumin-bound paclitaxel compared with polyethylated castor oil-based paclitaxel in women with breast cancer. *Journal of Clinical Oncology* 2005; **23**: 7794–803.

Emens LA, Cruz C, Eder JP, *et al.* Long-term Clinical Outcomes and Biomarker Analyses of Atezolizumab Therapy for Patients With Metastatic Triple-Negative Breast Cancer: A Phase 1 Study. *JAMA Oncol* 2019; **5**: 74–82.

Wasan H, Meade AM, Adams R, *et al.* Intermittent chemotherapy plus either intermittent or continuous cetuximab for first-line treatment of patients with KRAS wild-type advanced colorectal cancer (**COIN-B**): A randomised phase 2 trial. *Lancet Oncol* 2014; **15**: 631–9.

Overman MJ, McDermott R, Leach JL, *et al.* Nivolumab in patients with metastatic DNA mismatch repair-deficient or microsatellite instability-high colorectal cancer (**CheckMate 142**): an open-label, multicentre, phase 2 study. *Lancet Oncol* 2017; **18**: 1182–91.

Kopetz S, Grothey A, Yaeger R, *et al.* **BEACON CRC** Encorafenib, Binimetinib, and Cetuximab in BRAF V600E–Mutated Colorectal Cancer . *New England Journal of Medicine* 2019; **381**: 1632–43.

Azad NS, Gray RJ, Overman MJ, *et al.* Nivolumab Is Effective in Mismatch Repair-Deficient Noncolorectal Cancers: Results From Arm Z1D-A Subprotocol of the **NCI-MATCH** (EAY131) Study. *J Clin Oncol* 2020; **38**: 214–22.

Ghorani E, Kaur B, Fisher RA, *et al.* Pembrolizumab is effective for drug-resistant gestational trophoblastic neoplasia. *The Lancet* 2017; **390**: 2343–5.

Harrington KJ, Burtness B, Greil R, *et al.* Pembrolizumab With or Without Chemotherapy in Recurrent or Metastatic Head and Neck Squamous Cell Carcinoma: Updated Results of the Phase III **KEYNOTE-048** Study. *Journal of Clinical Oncology* 2023; **41**: 790–802.

Gandhi L, Rodríguez-Abreu D, Gadgeel S, *et al.* **KEYNOTE-189** Pembrolizumab plus Chemotherapy in Metastatic Non–Small-Cell Lung Cancer. *New England Journal of Medicine* 2018; **378**: 2078–92.

Reck M, Rodríguez-Abreu D, Robinson AG, *et al.* **KEYNOTE-024** Pembrolizumab versus Chemotherapy for PD-L1–Positive Non–Small-Cell Lung Cancer. *New England Journal of Medicine* 2016; **375**: 1823–33.

Soria J-C, Ohe Y, Vansteenkiste J, *et al.* **FLAURA** Osimertinib in Untreated EGFR -Mutated Advanced Non–Small-Cell Lung Cancer . *New England Journal of Medicine* 2018; **378**: 113–25.

Mok TS, Wu Y-L, Ahn M-J, *et al.* **AURA-3** Osimertinib or Platinum–Pemetrexed in EGFR T790M–Positive Lung Cancer . *New England Journal of Medicine* 2017; **376**: 629–40.

Denault MH, Kuang S, Shokoohi A, *et al.* Comparison of 2-Weekly Versus 4-Weekly Durvalumab Consolidation for Locally Advanced NSCLC Treated With Chemoradiotherapy: A Brief Report. *JTO Clin Res Rep* 2022; **3**. DOI:10.1016/J.JTOCRR.2022.100316.

Biesma B, Wymenga ANM, Vincent A, *et al.* Quality of life, geriatric assessment and survival in elderly patients with non-small-cell lung cancer treated with carboplatin-gemcitabine or carboplatin-paclitaxel: NVALT-3 a phase III study. *Ann Oncol* 2011; **22**: 1520–7.

Planchard D, Smit EF, Groen HJM, *et al.* Dabrafenib plus trametinib in patients with previously untreated BRAFV600E-mutant metastatic non-small-cell lung cancer: an open-label, phase 2 trial. *Lancet Oncol* 2017; **18**: 1307–16.

Planchard D, Besse B, Groen HJM, *et al.* Dabrafenib plus trametinib in patients with previously treated BRAFV600E-mutant metastatic non-small cell lung cancer: an open-label, multicentre phase 2 trial. *Lancet Oncol* 2016; **17**: 984–93.

Zhou H, Zeng C, Wei Y, Zhou J, Yao W. Duration of Chemotherapy for Small Cell Lung Cancer: A Meta-Analysis. *PLoS One* 2013; **8**. DOI:10.1371/JOURNAL.PONE.0073805.

Knispel S, Gassenmaier M, Menzies AM, *et al.* Outcome of melanoma patients with elevated LDH treated with first-line targeted therapy or PD-1-based immune checkpoint inhibition. *Eur J Cancer* 2021; **148**: 61–75.

Larkin J, Chiarion-Sileni V, Gonzalez R, *et al.* **CHECKMATE-067** Five-Year Survival with Combined Nivolumab and Ipilimumab in Advanced Melanoma. *New England Journal of Medicine* 2019; **381**: 1535–46.

Fennell DA, Ewings S, Ottensmeier C, *et al.* Nivolumab versus placebo in patients with relapsed malignant mesothelioma (**CONFIRM**): a multicentre, double-blind, randomised, phase 3 trial. *Lancet Oncol* 2021; **22**: 1530–40.

De Mestier L, Walter T, Brixi H, *et al.* Comparison of Temozolomide-Capecitabine to 5-Fluorouracile-Dacarbazine in 247 Patients with Advanced Digestive Neuroendocrine Tumors Using Propensity Score Analyses. *Neuroendocrinology* 2019; **108**: 343–53.

Kristeleit R, Lisyanskaya A, Fedenko A, *et al.* Rucaparib versus standard-of-care chemotherapy in patients with relapsed ovarian cancer and a deleterious BRCA1 or BRCA2 mutation (**ARIEL4**): an international, open-label, randomised, phase 3 trial. *Lancet Oncol* 2022; **23**: 465–78.

Davis ID, Martin AJ, Stockler MR, *et al.* Enzalutamide with Standard First-Line Therapy in Metastatic Prostate Cancer (**ENZAMET**). *New England Journal of Medicine* 2019; **381**: 121–31.

Sydes MR, Spears MR, Mason MD, *et al.* Adding abiraterone or docetaxel to long-term hormone therapy for prostate cancer: Directly randomised data from the **STAMPEDE** multi-arm, multi-stage platform protocol. *Annals of Oncology* 2018; **29**: 1235–48.

Atkins MB, Jegede OA, Haas NB, *et al.* Phase II Study of Nivolumab and Salvage Nivolumab/Ipilimumab in Treatment-Naive Patients with Advanced Clear Cell Renal Cell Carcinoma (HCRN GU16-260-Cohort A). *Journal of Clinical Oncology* 2022; **373**. DOI:10.1200/JCO.21.02938.

Motzer RJ, Tannir NM, McDermott DF, *et al.* Nivolumab plus Ipilimumab versus Sunitinib in Advanced Renal-Cell Carcinoma (**CheckMate 214**). *New England Journal of Medicine* 2018; **378**: 1277–90

Choueiri TK, Kluger H, George S, *et al.* **FRACTION-RCC**: nivolumab plus ipilimumab for advanced renal cell carcinoma after progression on immuno-oncology therapy. *J Immunother Cancer* 2022; **10**: e005780

Example of Interim Treatment Change Option produced by NHS England during the pandemic:

Interim treatment change options during the COVID-19 pandemic, endorsed by NHS England

The aim of the interim treatment changes are to allow for greater flexibility in the management of cancer during COVID-19 pandemic.

These interim treatment regimens are based on clinical opinion from members of the Chemotherapy Clinical Reference Group and cancer pharmacist and endorsed by NHS England and NHS Improvement.

The responsibility for using these interim treatment regimens lies entirely with the prescribing clinician, who must discuss the risks and benefits of interim treatment regimens with individual patients, their families and carers. All patients who start on an interim treatment during the COVID-19 pandemic should be allowed to continue the treatment until they and their clinician jointly decide it is appropriate to stop or to switch to a different treatment.

Treatment regimens will revert to the standard commissioned position once the emergency measures put in place to address the COVID-19 pandemic are no longer necessary.

These interim treatment changes to do not constitute NICE guidance.

| **Indication** | **Treatment changes** |
| --- | --- |
| General | - Give prophylactic daily granulocyte-colony stimulating factor (G-CSF) or a biosimilar PEGylated G-CSF to prevent neutropenic fever and reduce admissions (for example, for patients on chemotherapy regimens with a greater than 10% risk of neutropenic fever*)* - After an assessment of the risks and benefits to the patient, consider stopping:   - later-line palliative treatment to reduce the need for admission   - adjuvant therapy for low-risk patients, for example those with breast, lung or colorectal cancer, to reduce the need for immune-suppressive therapy |
| Breast cancer | - Suspend treatment with adjuvant bisphosphonates to reduce inpatient visits - Reduce the course of adjuvant trastuzumab treatment from 12 months to 6 months - Give pertuzumab plus trastuzumab for neo-adjuvant therapy, adjuvant therapy, locally recurrent or metastatic disease without chemotherapy to reduce the risk of   neutropenia |

|  | - Switch to oral capecitabine from intravenous taxanes with anti-HER2 therapies for metastatic disease to reduce the risk of neutropenia - Substitute albumin-bound paclitaxel (Abraxane) for paclitaxel or docetaxel to reduce toxicity and potential for admission |
| --- | --- |
| Colorectal cancer | - Allow intermittent treatment with chemotherapy regimens that contain cetuximab or panitumumab to reduce the need for immunosuppressive treatment |
| Non-small cell lung cancer | - Stop maintenance pemetrexed in combination with pembrolizumab to reduce treatment toxicity and risk of neutropenia - Allow pembrolizumab to be given as a single agent as a first-line treatment for squamous or non-squamous NSCLC and a PDL-1 score of less than 50% to reduce treatment toxicity and risk of neutropenia - Allow durvalumab be given 4 weekly in patients eligible for durvalumab following treatment with chemo- radiotherapy to reduce the number of hospital visits - Switch to carboplatin and paclitaxel from day 8 treatments such as gemcitabine and carboplatin and cisplatin and vinblastine |
| Small cell lung cancer | - Stop first-line chemotherapy for stage IV SCLC after 4 cycles to reduce hospital admission and risk of neutropenia |
| Melanoma | - Give oral therapy as first-line treatment for BRAF- positive patients in preference to immunotherapy to reduce admission for IV therapy - Stop immunotherapy doublet (ipilimumab and nivolumab) and switch to single agent nivolumab or pembrolizumab to reduce toxicity |
| Neuroendocrine tumours | - Give oral temozolomide and capecitabine instead of intravenous streptozocin and 5-fluorouracil to reduce toxicity and admissions for treatment |
| Ovarian cancer | - Give olaparib or other poly-ADP-ribose polymerase (PARP) inhibitors instead of chemotherapy plus maintenance PARP at first relapse for BRCA-positive PARP-naive patients to reduce admissions and risk of neutropenia |
| Renal cell cancer | - Stop first-line immunotherapy using nivolumab with ipilimumab in intermediate and poor risk groups, and |

|  | switch to either first-line single agent nivolumab or use oral therapy as first-line and nivolumab with ipilimumab as second-line therapies to reduce toxicity   - Use first- and second-line oral tyrosine kinase inhibitors and switch nivolumab from second- to third-line to delay use of IV immunotherapy (hospital visits) |
| --- | --- |
| Non-Hodgkin’s lymphoma | - Suspend subcutaneous rituximab maintenance to avoid patients attending hospital - Suspend subcutaneous obinutuzumab maintenance to avoid patients attending hospital |
| Myeloma | - Allow oral pomalidomide with dexamethasone as second- or third-line therapy instead of intravenous treatments in patients previously treated with lenalidomide to reduce the need for chemotherapy and reduce admissions and risk of neutropenia - Allow first-line lenalidomide and dexamethasone for transplant eligible myeloma patients in preference to regimens that require more hospital attendances and parenteral administrations to reduce toxicity of treatment and number of admissions required for treatment - Allow second^-^line lenalidomide and dexamethasone for patients who have not been previously treated with bortezomib |
